# Levels, sources, variations, and human health risk assessment of ^90^Sr and ^137^Cs in water and food around Sanmen Nuclear Power Plant (China) from 2011 to 2020

**DOI:** 10.3389/fpubh.2023.1136623

**Published:** 2023-02-22

**Authors:** Peng Wang, Shunfei Yu, Hua Zou, Xiaoming Lou, Hong Ren, Lei Zhou, Zhongjun Lai, Zhiqiang Xuan, Xiangjing Gao, Qiuliang Xu, Zhen Zhou, Yaoxian Zhao, Yiyao Cao

**Affiliations:** Institute of Occupational Health and Radiation Protection, Zhejiang Provincial Center for Disease Control and Prevention, Hangzhou, China

**Keywords:** radioactivity levels, Sanmen Nuclear Power Plant, radiation exposure, ^90^Sr, ^137^Cs, food, water, dose assessment

## Abstract

**Objectives:**

Radioactivity monitoring around nuclear facilities is crucial to provide important baseline data for effective detection of radioactive leakage to the environment. We aim to establish a baseline study for monitoring radioactive levels of ^90^Sr and ^137^Cs around Sammen Nuclear Power Plant (SNPP) and to assess their associated health impact on surrounding residents.

**Methods:**

In this study, we collected water and food samples around the SNPP from 2011 to 2020 and determined for ^90^Sr and ^137^Cs activity concentrations. We statistically analyzed the temporal trends of ^90^Sr and ^137^Cs and evaluated their radiation exposure to the local residents.

**Results:**

During this period, the activity concentrations of ^90^Sr and ^137^Cs varied within 1.2–9.9 mBq/L and 0.10–7.6 mBq/L in water, and 0.037–1.3 Bq/kg and 0.011–0.45 Bq/kg in food, respectively, with no significant seasonal variation trend.

**Conclusions:**

All reported activity concentrations of ^90^Sr and ^137^Cs were significantly lower than the recommended value of WHO and Chinese national standards. There is no indication of notable radioactive release into the study area due to the operation of SNPP during 2018–2020. The annual effective doses (*AEDs*) from the ingestion of ^90^Sr and ^137^Cs in water and food were well below the international permissible limits, indicating the radiation exposure around SNPP during 2011–2020 was kept at a safe level.

## 1. Introduction

Over the past few decades, the contribution of nuclear energy to the overall energy resources has increased rapidly worldwide. ^90^Sr and ^137^Cs are two recognized important fission products released from nuclear facility operations and nuclear accidents (1–3). These two radionuclides have relatively long physical half-lives (^90^Sr, t_½_ = 28.8 y and ^137^Cs, t_½_ = 30.2 y), and once released into the environment, they can be retained for a long time. In addition, ^90^Sr and ^137^Cs have high radiotoxicity, which can be enriched in bones and muscle tissues after entering organisms (4, 5). Therefore, ^137^Cs and ^90^Sr are highly essential artificial radionuclides to be taken into account in radiation risk assessment for the public and the environment (6–10). The digestion and assimilation of water and food is an important route for ^90^Sr and ^137^Cs to enter the human body. Therefore, systematic and continuous monitoring of ^90^Sr and ^137^Cs concentrations in water and food is crucial to ensure the radiation safety of the public, which is also a common practice globally for environmental radioactivity monitoring and radiological risk assessment around nuclear facilities (11–13).

Sanmen Nuclear Power Plant (SNPP) is one of the achievements of China's vigorous development of nuclear power, which uses the world's most advanced third-generation pressurized water reactor technology. SNPP covers a total area of 7.4 million square meters, with installation of six of 1.25 million kilowatt nuclear power units (AP1000). It began construction in 2009 and was commenced for operation in 2018. However, very few studies have reported the radioactivity levels nearby SNPP (14, 15), and no baseline data exist for the fission products such as ^137^Cs and ^90^Sr in water and food in the surrounding area. In this work, we report for the first time long-term monitoring results of ^90^Sr and ^137^Cs in water and food samples collected around SNPP form 2011 to 2020. The levels, temporal variations, and sources of ^90^Sr and ^137^Cs in the study area were studied, and the annual effective doses to the local public were estimated based on our monitoring data.

## 2. Materials and methods

### 2.1. The study region and sample collection

SNPP is located in Sanmen County, Taizhou City, Zhejiang Province, China, which is 171 km to the north of Hangzhou City, 83 km to the east of Ningbo City, 51 km to the west of Taizhou City, and 150 km to the south of Wenzhou City. During 2011–2020, all samples were collected within 30 km of the SNPP.

Sampling sites of surface water, factory water and tap water were selected in the distance of 5–10, 10–20, 20–30 km from SNPP based on the water source distribution and water supply characteristics of Sanmen County. The reservoir located 10.2 km away from SNPP is one of the main water sources for local residents, which was set as surface water sampling site. Sanmen County has a complete water supply system, and the tap water comes from the municipal water supply company. Factory water sampling site was set at the water plant 6.0 km away from SNPP, and tap water sampling site was set at the Sanmen County Center for Disease Control and Prevention, which is 22.7 km away from SNPP. In order to reflect the time trend and seasonal changes, water samples were collected in the wet season (May) and dry season (October) in each year.

**Figure 1:**
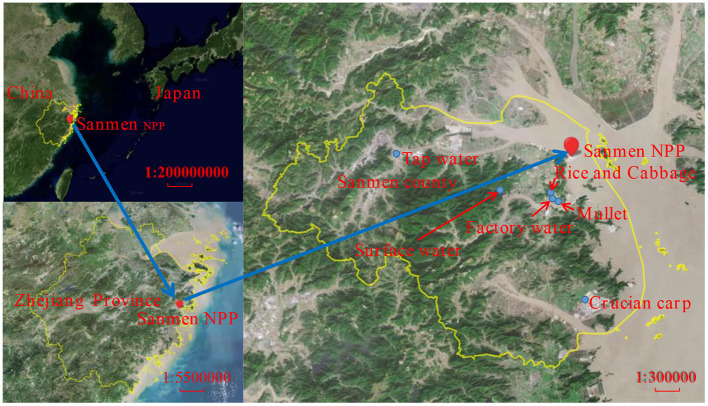
Sampling sites for water and food around SNPP in this study. The map was produced using software MapInfo Professional using data from https://bajiu.cn/ditu/.

According to the dietary habits of local residents, four typical types of food were selected in this work: rice, cabbage, crucian carp and mullet, which were collected in the harvest season every year. The sampling information of water and food samples in this work are summarized in Figure 1 and Table 1.

**Table 1:** Water and food samples collected around SNPP in this study.

**Sample category**	**Type or species**	**Distance from SNPP (km)**	**Location (coordinates)**	**Sample volume/weight**	**Radionuclide analyzed**	**Number of subsamples collected each time**
Water	Surface water	10.2	29°03′24″(N) 121°33′02″(E)	70 L	^90^Sr, ^137^Cs	4
	Factory water	6.0	29°02′48″(N) 121°37′41″(E)	70 L	^90^Sr, ^137^Cs	4
	Tap water	22.7	29°06′43″(N) 121°24′03″(E)	70 L	^90^Sr, ^137^Cs	4
Food	Rice	6.0	29°03′45″(N) 121°36′55″(E)	20 kg	^90^Sr, ^137^Cs	4
	Cabbage	6.0	29°03′45″(N) 121°36′55″(E)	10 kg	^90^Sr, ^137^Cs	4
	Crucian carp	18.9	28°55′44″(N) 121°39′59″(E)	10 kg	^90^Sr, ^137^Cs	4
	Mullet	6.6	29°02′41″(N) 121°38′01″(E)	10 kg	^90^Sr, ^137^Cs	4

The prevailing wind direction of SNPP region was north and north-northwest (16). In this work, all sampling sites were located in the south or southwest of SNPP (except for tap water sampling site, located in the west of SNPP), which were located in the leeward direction of the prevailing wind direction. In addition, tap water was transported through pipelines, the influence of wind direction was considered negligible. Therefore, we believe the sampling sites in this work were representative.

### 2.2. Reagents and materials

Anhydrous ethanol (CH_3_CH_2_OH) (Analytical grade, Anhui Antell Food Co., LTD.), acetic acid (CH_3_COOH) (Analytical grade, Tianjin Damao Chemical Reagent factory), sodium hydroxide (NaOH), hydrochloric acid (HCl), sodium sulfide (Na_2_S), nitric acid (HNO_3_), ammonium phosphomolybdate [(NH_4_)_3_PMo_12_O_40_], hydrogen peroxide (H_2_O_2_) (Analytical grade, Sinopharm Chemical Reagent Co., LTD.), ammonia (NH_3_·H_2_O) [Analytical grade, Aladdin Reagent (Shanghai) Co., LTD], 2- (2-ethylhexyl) phosphoric acid (Analytical grade, 60~80 mesh, Beijing Research Institute of chemical Engineering Metallurgy). ^90^Sr-^90^Y standard solution (9.78 Bq/g of ^90^Sr in 0.1 mol/L nitric acid) and ^137^Cs standard solution (1.47 Bq/g of ^137^Cs in 0.1 mol/L nitric acid) were purchased from National Institute of Metrology, China. ^90^Sr-^90^Y reference source was purchased from Beijing Research Institute of Chemical Engineering Metallurgy, with source intensity (surface particle number/2π·min) of 1.20 × 10^3^.

### 2.3. Sample preparation and analysis

**Figure 2:**
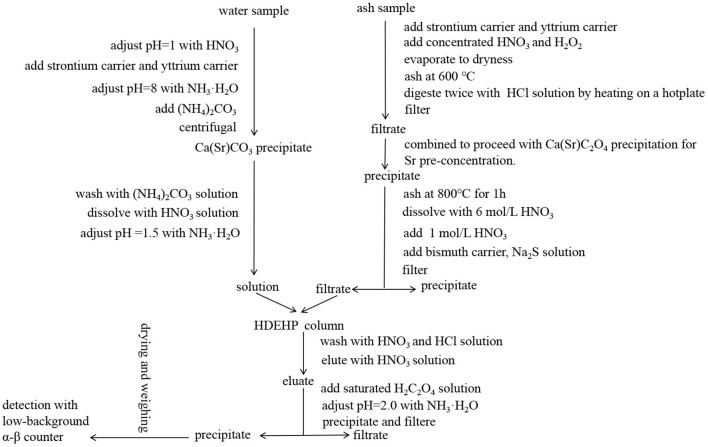
Radioanalytical procedure used in this work for ^90^Sr determination in water and food samples.

**Figure 3:**
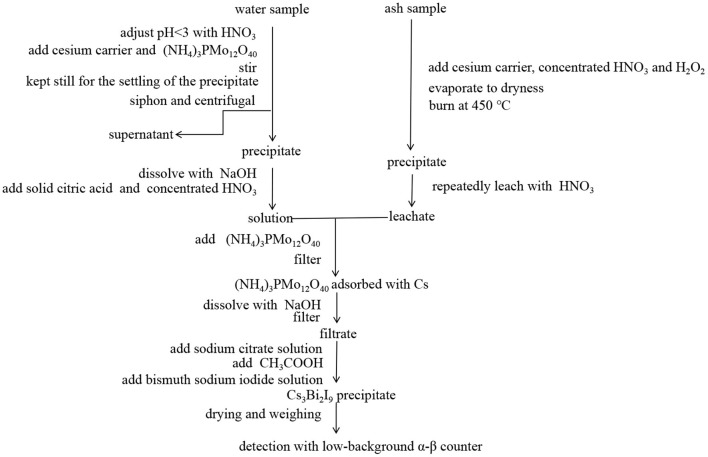
Radioanalytical procedure used in this work for ^137^Cs determination in water and food samples.

After cleaning (washing the sample with water), each fresh food sample was weighted and loaded into a microwave furnace for drying, carbonization and ashing under programmed gradient heating conditions (17). The detailed microwave heating conditions for different types of samples are shown in Table 2. The schematic radioanalytical procedures used in this work for ^90^Sr and ^137^Cs determination are demonstrated in Figures 2, 3, respectively.

**Table 2:** Programmed heating conditions of microwave-ashing furnace for food sample pre-treatment in this study.

**Food species**	**Temperature (°C)**	**Duration (h)**	**Food species**	**Temperature (°C)**	**Duration (h)**
Cabbage	98	0.5	Crucian carp	127	2
	240	3		230	10
	320	1		350	2
	450	4		450	12
Mullet	105	1	Rice	150	12
	230	10		270	12
	300	2		400	5
	450	10		450	5

To analyze ^90^Sr concentrations in water, 30 L of water sample was taken and adjusted to pH = 1.0 with HNO_3_. After adding 2.0 mL of 50 mg/mL strontium carrier and 1.0 mL of 20 mg/mL yttrium carrier, the pH was adjusted to 8.0 with addition of NH_3_·H_2_O. 240 g of (NH_4_)_2_CO_3_ were added to form Ca(Sr)CO_3_ precipitate, which was separated by centrifugation (twice at 4000 r/min for 5 min). The precipitate was washed with 1% (NH_4_)_2_CO_3_ solution and dissolved in 6 mol/L of HNO_3_. After adjusting the pH to 1.5 with NH_3_·H_2_O, the solution was loaded onto a chromatographic column (1.0 cm inner diameter × 14 cm length) packed with di-(2-ethylhexyl) phosphate (HEDHP) at a flow rate of 2 ml/min. The column was sequentially washed with 10 ml of 1.5 mol/L HNO_3_, 30 ml of 1.0 mol/L HCl and 30 ml of 1.5 mol/L HNO_3_ at 2 ml/min. Yttrium (Y) was eluted with 30 ml of 6 mol/L HNO_3_ at 1 ml/min, the eluate was collected. The starting and ending times of the column separation were recorded for decay correction of ^90^Y. 5 mL of saturated H_2_C_2_O_4_ solution was added into the eluate. The sample solution was adjusted to pH = 2.0 with ammonia, and heated in a water bath at 80 °C for 30 min to precipitate Y as yttrium oxalate Y_2_(C_2_O_4_)_3_, then cooled to room temperature. Yttrium oxalate precipitate was filtered through filter paper and washed successively with 10 mL of 1% H_2_C_2_O_4_, 10 mL of water and 10 mL of CH_3_CH_2_OH, then dried at 45–50°C until the weight was constant. The chemical recovery of Y was calculated using the weight of obtained yttrium oxalate according to the molecular formula of Y_2_(C_2_O_4_)_3_·9H_2_O. The yttrium oxalate precipitate was detected by low-background α-β counter (BH1217II, China National Nuclear Corporation, China; LB790, Berthold Technologies, Germany), with each sample counted for 10 cycles and 100 min. for each cycle (18, 19).

To analyze ^90^Sr concentrations in food, 10g of ash sample was spiked with 2.0 mL of 50 mg/mL strontium carrier and 1.0 mL of 20 mg/mL yttrium carrier. After adding 10 mL of concentrated HNO_3_ and 6 mL of H_2_O_2_, the sample was evaporated to dryness and ashed at 600 °C until a complete decomposition of organic substance. The sample was digested twice with 40 mL of 2 mol/L HCl solution by heating on a hotplate. The leachate was filtered and combined to proceed with Ca(Sr)C_2_O_4_ precipitation for Sr pre-concentration. The precipitate was ashed in a muffle furnace at 800°C for 1h, dissolved with a few milliliter of 6 mol/L HNO_3_. 35 mL of 1 mol/L HNO_3_ was added to completely dissolve the precipitate. After the addition of 0.5 mL of bismuth carrier (20 mg/mL), 0.4 mL of 0.3mol/L Na_2_S solution was dropped to generate black Bi_2_S_3_ precipitate to removal of interfering ions such as ^210^Bi and rare earth elements. The solution was filtered with filter paper and the filtrate (about 60 mL) was collected for further chromatographic purification using HDEHP as described above (19, 20).

To analyze ^137^Cs concentrations in water, 30 L of water sample was adjusted to pH < 3.0 with HNO_3_. 1.0 mL of 20 mg/mL cesium carrier and 6 g of (NH_4_)_3_PMo_12_O_40_ were added to the sample, which was stirred by electric mixer, then kept still for the settling of the precipitate. The supernatant was discarded by siphon, and the remaining precipitate was centrifuged at 3500 r/min for 5 min to remove the liquid phase. The precipitate was dissolved in 60 mL of 2.0 mol/L NaOH, 10 g of solid citric acid and 10 ml of concentrated HNO_3_ were added. After adding 0.8 g of (NH_4_)_3_PMo_12_O_40_, the sample was filtrated to separate the (NH_4_)_3_PMo_12_O_40_ on which cesium was adsorbed. 10 mL of 2.0 mol/L NaOH were added to dissolve (NH_4_)_3_PMo_12_O_40_ and the solution was filtered again. 5 ml of sodium citrate solution (mass ratio 30%) was added, followed by addition of 2 ml of glacial CH_3_COOH and 2.5 ml of bismuth sodium iodide solution (mass ratio 42%). The sample was cooled in an ice water bath to finally precipitate cesium as Cs_3_Bi_2_I_9_. Cs_3_Bi_2_I_9_ precipitate was dried and weighed to obtain the chemical yield of Cs, and then detected by low-background α-β counter (BH1217II, China National Nuclear Corporation, China; LB790, Berthold Technologies, Germany). Each sample was counted for 10 cycles with 100 min. for each cycle (21, 22).

To analyze ^137^Cs concentrations in food, samples were stored for 1 month to allow ^131^I to decay completely. Each dried food sample was screened by gamma spectrometry to eliminate interferences to the ^137^Cs measurements from other short-lived radiocesium (i.e., ^134^Cs, ^136^Cs, and ^138^Cs). To 10 g of ash sample, 1.0 mL of 20 mg/mL cesium carrier, 10 mL of concentrated HNO_3_ and 3 mL of H_2_O_2_ were added. After evaporating to dryness, the sample was burned in a muffle furnace at 450°C, then repeatedly leached with 1.5 mol/L HNO_3_. 0.8 g of (NH_4_)_3_PMo_12_O_40_ were added to the leachate and the sample was thereafter processed following the same procedure as described above (22, 23).

The main factors affecting the uncertainty of the measurement results of ^90^Sr and ^137^Cs in water and food samples were the uncertainties of β radioactivity measurement (counting statistics), instrument detection efficiency, chemical yield and sample quality. The typical relatively uncertainties for ^90^Sr and ^137^Cs activity concentrations obtained in this work were at the level of 5–27%.

### 2.4. Dose assessment

In order to carry out dose assessment for residents around SNPP, Zhejiang Center for Disease Control and Prevention conducted a food consumption survey of residents in Taizhou, Zhejiang Province from 2015 to 2017. Three counties in Taizhou city, Zhejiang province were chosen for this survey, using multi-stage stratified proportional to population cluster random sampling method, i.e., each site from three villages and towns (streets), each street draw two villages (neighborhood committees), every village (neighborhood committees) extract 50 families, the permanent members of each selected family (resident for at least 6 months, resident aged 3 years and above) were identified as the respondents after signing an “informed consent”.

The recorded data of the consumption frequency and average consumption amount of rice, cabbage, crucian carp, mullet and related products were collected by face-to-face survey (using the method of 24 h retrospective for 3 consecutive days). The questionnaire was based on the recommendations from China National Center for Food Safety Risk Assessment. The dietary information of all respondents under the age of 18 was provided by their guardians. According to the survey results, the average consumption of four types of food in Taizhou city, Zhejiang Province was calculated for people of different ages (grouping criteria: 3–12 years old, 12–18 years old and more than 18 years old).

The annual effective dose (*AED*) due to the ingestion of ^137^Cs and ^90^Sr in food was estimated using the following equation:


$$\begin{array}{l} AED=I_{\text{Sr}}e{\left(g\right)}_{\text{Sr}}+I_{\text{Cs}}e{\left(g\right)}_{\text{Cs}} \end{array}$$

Where, *I*_*Sr*_ or *I*_*Cs*_ is the annual intake of ^90^Sr or ^137^Cs (Bq/year) from food. *e*(*g*)_*Sr*_ or *e*(*g*)_*Cs*_ is the age-dependent dose conversion coefficient for ingestion of ^90^Sr or ^137^Cs. The corresponding *e*(*g*)_*Sr*_ and *e*(*g*)_*Cs*_ values used in this study were according to Chinese national standards (24), namely, 6.0 × 10^−8^ Sv/Bq and 1.0 × 10^−8^ Sv/Bq, 8.0 × 10^−8^ Sv/Bq and 1.3 × 10^−8^ Sv/Bq, 2.8 × 10^−8^ Sv/Bq and 1.3 × 10^−8^ Sv/Bq, for people with age of 3~12 years, 12~18 years and more than 18 years, respectively.

### 2.5. Statistical analysis

SPSS 25.0 software was used for statistical analysis, and Mann–Whitney *U*-test was used to compare the radioactive activity concentration levels of water. *p* < 0.05 was considered as statistically significant.

## 3. Results and discussion

### 3.1. Activity concentrations of ^90^Sr and ^137^Cs in waters

**Figure 4:**
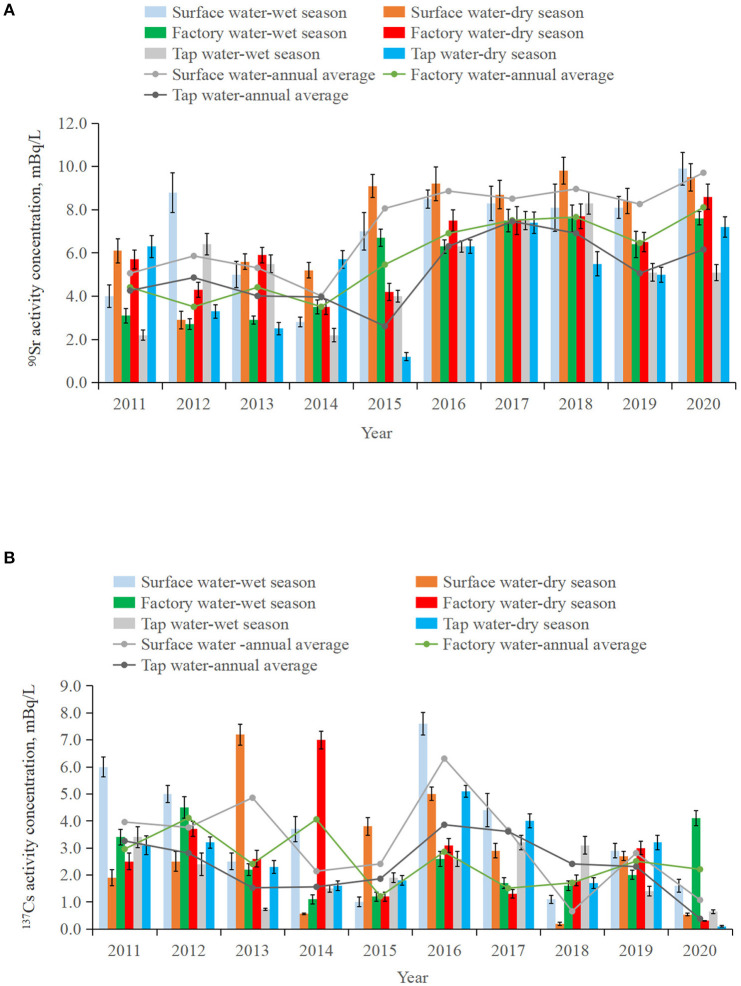
Variation of ^90^Sr **(A)** and ^137^Cs **(B)** activity concentrations in surface, factory and tap water collected around SNPP during wet and dry seasons in 2011–2020.

The time series of ^90^Sr and ^137^Cs activity concentrations in water samples collected around SNPP from 2011 to 2020 are shown in Figure 4. Statistical analysis indicated that, ^90^Sr and ^137^Cs activity concentrations in all types of water had no significant difference between the wet season and dry season in each year (*p* > 0.05). The monitoring results of ^90^Sr and ^137^Cs vary within ranges of (2.8 ± 0.2)–(9.9 ± 0.8) mBq/L and (0.19 ± 0.05)–(7.6 ± 0.4) mBq/L, (2.7 ± 0.3)–(8.6 ± 0.6) mBq/L and (0.30 ± 0.02)–(7.0 ± 0.3) mBq/L, (1.2 ± 0.2)–(8.3 ± 0.5) mBq/L and (0.10 ± 0.02)–(5.1 ± 0.2) mBq/L, for surface water, factory water and tap water, respectively. Compared to the concentration limits recommended by WHO (10 Bq/L for ^90^Sr and 10 Bq/L for ^137^Cs), the activity concentrations of ^90^Sr and ^137^Cs in water samples around SNPP from 2011 to 2020 were 3-4 orders of magnitude lower (25).

The annual mean activity concentrations of ^90^Sr in all three types of waters showed similar temporal changes, namely, notable increases are observed during 2016–2020 from relatively low and stable levels in 2011–2015 (Figure 4A). Different from ^90^Sr, activity concentrations of ^137^Cs in the three types of waters were more fluctuated during 2011–2020 (Figure 4B). For example, the difference between the maximum and minimum annual mean activity concentrations of ^137^Cs reached 3.4 times in factory water, and more than 9 times in tap water and surface water. In the case for ^90^Sr, the difference was <2.9 times. This might be related to the higher solubility and mobility of Sr in water, whereas Cs is more particle reactive in the natural environment (26). The highest ^137^Cs annual mean activity concentrations were seen in 2016 for both surface water and tap water.

Compared with tap water, the annual mean activity concentrations of ^90^Sr in surface water were generally higher (Figure 4A). Statistical analysis also confirmed this difference (*p* < 0.05). This phenomena was consistent with our previous findings (27), which should be related to additional water treatment processes (e.g. coagulation, filtration and precipitation) from surface water to tap water.

Table 3 showed the activity concentrations of ^90^Sr and ^137^Cs in waters from different regions of the world. The values of waters around SNPP were at background levels and at the same order of magnitude as radioactivity levels in different waters around the globe (11, 27–37).

**Table 3:** Comparison of ^90^Sr and ^137^Cs activity concentrations in waters from different regions of the world.

**Location**	**Sample type (Sampling time)**	**Activity concentration**	
	^90^ **Sr (mBq/L)**	^137^ **Cs (mBq/L)**
Poland (11)	Surface water (since 1994)	3.92 ± 0.40	4.49 ± 2.00
Qinshan Nuclear Power Plant, China (27)	Surface water (2012–2019)	4.3–11.1	0.9–7.0
	Tap water (2012–2019)	5.0– 8.2	1.4– 4.2
Greenland (28)	Terrestrial freshwater (1999–2001)	1.62–5.17	0.27– 2.41
Ninghai county, Ningbo city, China (29)	Drinking water (2013)	4.76– 9.02	2.23– 9.32
Shandong, China (30)	Lake water (/)	5.5	0.76
Yangtze River, China (31)	River water (2017–2018)	1.200– 6.030	/
Haiyan county, Jiaxing City, China. (32)	Underground water (1988 and 1991)	3.9	0.6
Qinshan Nuclear Power Plant, China (33)	Terrestrial freshwater (1992–2005)	4.4 ± 1.7	0.3 ± 0.1
Spain (34)	River water (/)	0.6–21.3	<0.17–1.4
Milano, Italy (35)	Tap water (since late 1980s)	/	<0.5
Cuban (36)	Spring water (/)	3.4 ± 0.7	<4
Tokyo, Japan (37)	Tap water (2011)	<0.4 (Bq/kg)	0.95–4.27 (Bq/kg)
Sanmen Nuclear Power Plant, China (this work)	Surface water (2011–2020)	2.8– 9.9	0.19–7.6
	Factory water (2011–2020)	2.7–8.6	0.30–7.0
	Tap water (2011–2020)	1.2– 8.3	0.10–5.1

### 3.2. Activity concentrations of ^90^Sr and ^137^Cs in foods

**Figure 5:**
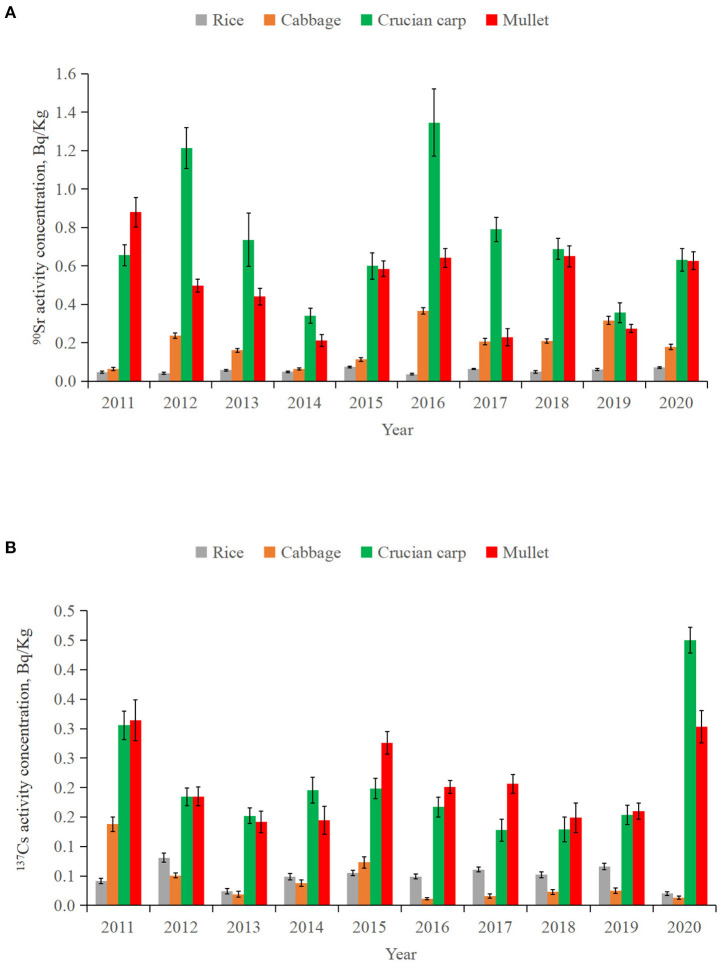
Activity concentrations of ^90^Sr **(A)** and ^137^Cs **(B)** in different food samples (rice, cabbage, crucian carp and mullet) around SNPP form 2011 to 2020.

Figure 5 shows the activity concentrations of ^90^Sr and ^137^Cs in different types of food samples around SNPP from 2011 to 2020. The variations in activity concentrations of ^90^Sr and ^137^Cs in all four types of food showed no significant inter-annual trend. The activity concentrations of ^90^Sr and ^137^Cs in rice, cabbage, crucian carp and mullet were (0.037 ± 0.004)–(0.075 ± 0.004) Bq/kg fresh weight (f. w.) and (0.020 ± 0.003)–(0.081 ± 0.008) Bq/kg f. w., (0.065 ± 0.005) – (0.37 ± 0.02) Bq/kg f. w. and (0.011 ± 0.002)–(0.14 ± 0.01) Bq/kg f. w., (0.34 ± 0.04)–(1.3 ± 0.2) Bq/kg f. w. and (0.13 ± 0.02)–(0.45 ± 0.02) Bq/kg f. w., (0.21 ± 0.03)–(0.88 ± 0.08) Bq/kg f. w. and (0.14 ± 0.02)–(0.31± 0.04) Bq/kg f. w., respectively (Table 4). Comparing to the limits of ^90^Sr and ^137^Cs activity concentrations in grain (96 and 260 Bq/kg), vegetables (77 and 210 Bq/kg) and fish (290 and 800 Bq/kg) recommended by Chinese national standard (38), the activity concentrations of ^90^Sr and ^137^Cs in food samples around SNPP were significantly lower.

**Table 4:** Activity concentrations of ^137^Cs and ^90^Sr and ^137^Cs/^90^Sr activity ratios in food samples around SNPP form 2011 to 2020.

**Year**		**Rice**			**Cabbage**			**Crucian carp**			**Mullet**		
		^90^ **Sr Bq/Kg**	^137^ **Cs Bq/Kg**	^137^**Cs/** ^90^**Sr**	^90^ **Sr Bq/kg**	^137^ **Cs Bq/kg**	^137^**Cs/** ^90^**Sr**	^90^ **Sr Bq/kg**	^137^ **Cs Bq/kg**	^137^**Cs/** ^90^**Sr**	^90^ **Sr Bq/kg**	^137^ **Cs Bq/kg**	^137^**Cs/** ^90^**Sr**
2011	value	0.048	0.041	0.86	0.065	0.14	2.13	0.66	0.31	0.47	0.88	0.31.	0.36
	sd	0.006	0.005	0.02	0.008	0.01	0.13	0.06	0.02	0.004	0.08	0.04	0.009
2012	value	0.042	0.081	1.94	0.24	0.051	0.22	1.2	0.18	0.15	0.50	0.19	0.37
	sd	0.005	0.008	0.03	0.01	0.004	0.004	0.1	0.02	0.001	0.03	0.02	0.008
2013	value	0.058	0.024	0.41	0.16	0.019	0.12	0.74	0.15	0.21	0.44	0.14	0.32
	sd	0.004	0.004	0.04	0.01	0.005	0.02	0.14	0.01	0.02	0.04	0.02	0.01
2014	value	0.049	0.049	1.00	0.065	0.038	0.58	0.34	0.20	0.58	0.21	0.14	0.68
	sd	0.005	0.006	0.02	0.005	0.006	0.04	0.04	0.02	0.02	0.03	0.02	0.03
2015	value	0.075	0.055	0.74	0.11	0.073	0.65	0.60	0.20	0.33	0.59	0.28	0.47
	sd	0.004	0.005	0.02	0.01	0.010	0.03	0.07	0.02	0.01	0.04	0.02	0.01
2016	value	0.037	0.049	1.32	0.37	0.011	0.031	1.3	0.17	0.12	0.64	0.20	0.31
	sd	0.004	0.004	0.04	0.02	0.002	0.003	0.2	0.02	0.006	0.05	0.01	0.007
2017	value	0.064	0.061	0.96	0.21	0.016	0.078	0.79	0.13	0.16	0.23	0.21	0.92
	sd	0.004	0.004	0.01	0.02	0.004	0.011	0.06	0.02	0.01	0.04	0.02	0.11
2018	value	0.049	0.052	1.08	0.21	0.023	0.11	0.69	0.13	0.19	0.65	0.15	0.23
	sd	0.006	0.005	0.05	0.01	0.004	0.01	0.06	0.02	0.02	0.06	0.02	0.02
2019	value	0.061	0.066	1.09	0.32	0.025	0.079	0.36	0.15	0.43	0.28	0.16	0.58
	sd	0.005	0.005	0.01	0.02	0.005	0.010	0.05	0.02	0.02	0.02	0.01	0.008
2020	value	0.071	0.020	0.28	0.18	0.013	0.069	0.63	0.45	0.71	0.63	0.30	0.48
	sd	0.004	0.003	0.03	0.02	0.003	0.009	0.06	0.02	0.03	0.05	0.03	0.01
Min.	value	0.037	0.020	0.28	0.065	0.011	0.031	0.34	0.13	0.12	0.21	0.14	0.23
	sd	0.004	0.003	0.03	0.005	0.002	0.003	0.04	0.02	0.006	0.03	0.02	0.02
Max.	value	0.075	0.081	1.94	0.37	0.14	2.13	1.3	0.45	0.71	0.88	0.31	0.92
	sd	0.004	0.008	0.03	0.02	0.01	0.13	0.2	0.02	0.03	0.08	0.04	0.11
Average	value	0.055	0.050	—	0.19	0.041	—	0.73	0.21	—	0.50	0.21	—
	sd	0.012	0.018	—	0.10	0.039	—	0.32	0.10	—	0.22	0.07	—

The activity concentrations of ^90^Sr varied greatly with food species (Figure 5A), with mean activity concentrations over the 10 years generally decreasing following the order of rice (0.055 ± 0.012 Bq/kg f. w.) < cabbage (0.19 ± 0.01 Bq/kg f. w.) < mullet (0.50 ± 0.22 Bq/kg f. w.) < crucian carp (0.73 ± 0.32 Bq/kg f. w.) (Table 4). In contrast to rice, other three types of food showed significant inter-annual variations in activity concentrations of ^90^Sr during 2011–2020. The difference between maximum and minimum of ^90^Sr activity concentrations was 5.7, 3.8, and 4.2 times in cabbage, crucian carp and mullet, respectively. The highest ^90^Sr values were both found in 2016 for cabbage and crucian carp, and in 2011 for mullet.

Different from ^90^Sr, the mean activity concentrations of ^137^Cs were comparable between rice (0.050 ± 0.018 Bq/kg f. w.) and cabbage (0.041 ± 0.039 Bq/kg f. w.), and between crucian carp (0.21 ± 0.10 Bq/Kg f. w.) and mullet (0.21 ± 0.07 Bq/Kg f. w.), with much lower levels in the former pair than the latter. These characteristics might be related to the different environmental conditions for the growth of these biota and different physiological metabolisms for ^90^Sr and ^137^Cs uptake and excretion among different biological species (4, 5).

Table 5 lists the activity concentrations of ^90^Sr and ^137^Cs in foods from different regions in the world. The values obtained in this study were at the same order of magnitude as ^137^Cs and ^90^Sr activity concentrations in food samples from other areas reported earlier (27, 29, 36, 39–49).

**Table 5:** Comparison of activity concentrations of ^137^Cs and ^90^Sr in foods from different regions of the world.

**Location**	**Sample type (Sampling time)**	**Activity concentration**	
		^90^ **Sr (Bq/kg)**	^137^ **Cs (Bq/kg)**
Qinshan Nuclera Power Plant, China (27)	Rice, Salsola, Mullet, Crucian carp (2012-2019)	0.04– 1.3	0.02– 0.6
Ninghai distict, Ningbo city, China (29)	Green vegetable, Pakchoi, Chub, Rice, Wheat (2013)	0.125– 0.64	0.021 (Only green vegetable)
Cuban (30)	Vegetables, Fish (/)	0.0034– 0.28	<0.004– 0.0043
Tianwan Nuclear Power Plant, China (39)	Rice, Chinese cabbage, wheat (March 2000 to April 2002)	0.024 - 0.23	0.009–0.033
Vicinity of Tianwan Nuclear Power Plant, China (40)	Rice, Chinese cabbage, Marine fish (2009-2010)	/	2.5– 12
Vicinity of Hongyanhe Nuclear Power Plant, China (41)	Cereals, Vegetables and fruits, meat, fish and shrimp (2009)	/	0.5–11.1
Vicinity of Ningde Nuclear Power Plant, China (42)	Rice, Vegetables, Marine fish, Freshwater fish (2013-2017)	<0.017– 0.677	/
Vicinity of Hongyanhe Nuclear Power Plant, China (43)	Vegetables and fruits, Cereals, Seafood (2013-2020)	0.005–0.428 (Only cereals and seafood)	0.005– 0.024
Hangzhou, China (44)	Rice, Salsola, Kelp, Hairtail, Crucian carp (2012~2019)	0.023–1.3	MDC - 0.29
Austrian (45)	Cereals, Cabbage, Freshwater fish (1997)	0.09– 0.12	0.08– 0.15
Kola Peninsula, Russia (46)	Freshwater fish, Marine fish (1998-1999)	/	1.4– 28
Amchitka Island, Alaska (47)	Marine fish (2004)	/	0.10– 0.32
Niigata, Japan (48)	Undaria pinnatifida(1999-2007)	<0.016– 0.036	0.034– 0.079
Mayak Industrial Association (49)	Wheat, Cabbage, Carrots(2008-2010)	0.029– 0.24	0.019–0.15
Sanmen Nuclear Power Plant, China (this work)	Rice, Cabbage, Mullet, Crucian carp (2011-2020)	0.037–1.3	0.011–0.45

### 3.3. Sources of ^90^Sr and ^137^Cs in the study region

**Figure 6:**
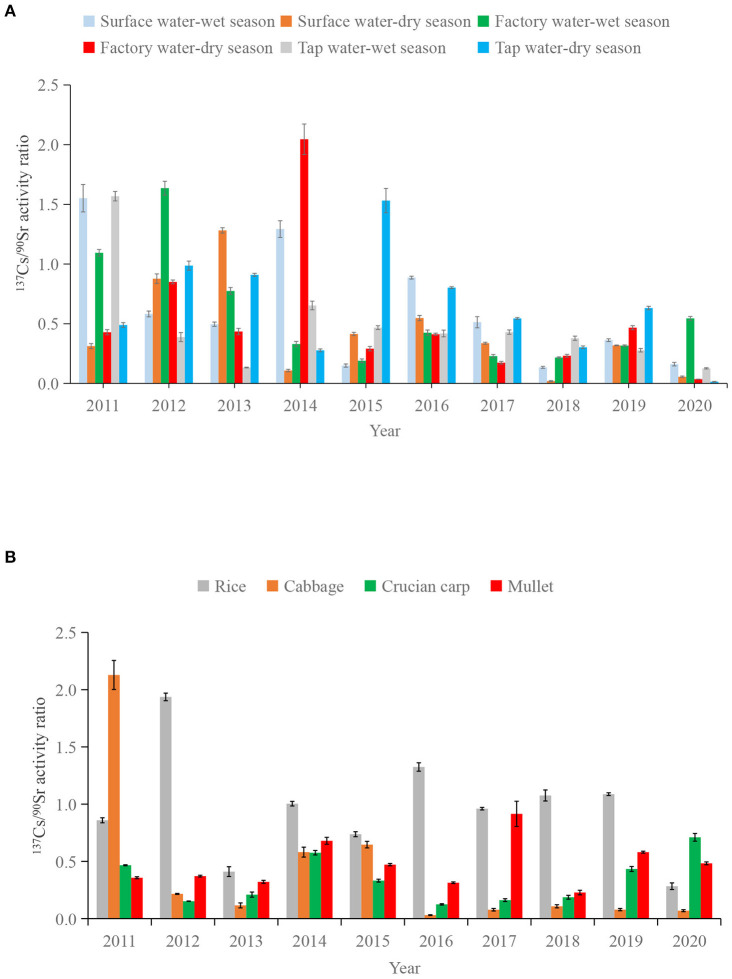
^137^Cs/^90^Sr activity ratios in water **(A)** and food **(B)** samples during 2011–2020.

Figure 6 shows the time series diagram of ^137^Cs/^90^Sr activity ratios in the water and food samples investigated from 2011 to 2020. The activity ratios of ^137^Cs/^90^Sr in surface water, factory water and tap water were in the ranges of (0.020 ± 0.004)–(1.55 ± 0.12), (0.035 ± 0.001)–(2.05 ± 0.13) and (0.014 ± 0.002)–(1.57 ± 0.04), respectively. The activity ratios of ^137^Cs/^90^Sr in rice, cabbage, crucian carp and mullet ranged within (0.28 ± 0.03)–(1.94 ± 0.03), (0.031 ± 0.003)–(2.13 ± 0.13), (0.12 ± 0.006)–(0.71 ± 0.03) and (0.23 ± 0.02)–(0.92 ± 0.11), respectively (Table 4).

The main pollution source of ^90^Sr and ^137^Cs in surface environment would be the global fallout from the atmospheric nuclear weapon tests conducted in 1950–1980s (50, 51). In the study region, in addition to the contamination caused by global fallout, there was also the possibility of radioactive fallout from the operation of local and regional nuclear power plants, e.g., SNPP and Qinshan NPP. Other pollution source, e.g., Chernobyl nuclear accident, was deemed to have contributed little to the quantity of ^90^Sr and ^137^Cs in the western North Pacific (52, 53). In addition, there were no evidences showing that Fukushima nuclear accident has significant impact on China. So far, no significant release of radioactivity has been reported from SNPP and Qinshan NPP. Therefore, ^90^Sr and ^137^Cs detected in water and food around SNPP should reflect the global radioactive fallout levels.

^137^Cs/^90^Sr activity ratio has been used in order to obtain evidence to clarify the origin of these radionuclides (54, 55). The activity ratio of ^137^Cs/^90^Sr from the global fallout deposition was estimated to be about 1.6 (51). Much higher ^137^Cs/^90^Sr activity ratios have been reported for the local fallout from the Chernobyl (up to 250) (56) and Fukushima (up to 1000) (2, 57) nuclear accidents. Our results showed that the ^137^Cs/^90^Sr activity ratios in water and food around SNPP were lower than the global fallout signal, except for individual samples. This may be related to the fact that ^90^Sr deposited in the soil is easier to migrate to crops and water, because of higher solubility and mobility of ^90^Sr in the surface environment (58). In our previous studies,the ^137^Cs/^90^Sr activity ratios in food and water samples form Qinshan NPP (China) (water: <1.2, food: <1.2) (27) and Hangzhou [water: <1.5(except for individual samples), food: <0.72] (44) were similar to this study, both lower than the global fallout signal.

In this study, the highest activity ratio in surface water occurred in wet season of 2011 (1.55 ± 0.12), which was 5 times higher than that in dry season (0.31 ± 0.02) in the same year, and 2.7 times higher than that in wet season of 2012 (0.58 ± 0.02). This might indicate additional radioactive input in the study area during the wet season of 2011. In addition, the maximum activity ratio of cabbage and rice appeared in 2011 and 2012 respectively, which further proved the possibility of our speculation (Figure 4). However, more in-depth investigation would be needed to clarify this potential source input.

In addition, after the operation of SNPP in 2018, activity ratios of ^137^Cs/^90^Sr in either food or water samples during 2018–2020 were not increased compared to earlier year. This indicates that radioactive substances released during the operation of SNPP in 2018–2020 were negligible.

### 3.4. Dose assessment

According to the annual average food intake of Sanmen County residents (Located in Taizhou City), as shown in Table 6, the mean *AED* values from 2011 to 2020 were calculated to be 7.2 × 10^−7^, 1.3 × 10^−6^ and 6.8 × 10^−7^ Sv/y, respectively, for people of 3-12 years old, 12-18 years old and more than 18 years old. They were significantly lower than the reference value recommended by Chinese national standards (7.84 × 10^−4^ Sv/y) (38), indicating that radioactivity concentrations of ^90^Sr and ^137^Cs in food from Sanmen County were at safe levels from 2011 to 2020.

**Table 6:** Annual average food intake by Sanmen residents.

**Group**	**Rice (kg)**	**Cabbage (kg)**	**Mullet (kg)**	**Crucian carp (kg)**
3–12 years old	127.266	1.27	5.74	0.778
12–18 years old	196.933	0.93	5.31	0.635
More than 18 years old	214.055	1.95	10.09	1.389

## 4. Conclusions

This work provides a long-term and systematic study on the levels, variations, and sources of ^90^Sr and ^137^Cs in water and food around SNPP from 2011 to 2020, with dose assessment for the local public. The activity concentrations of ^90^Sr and ^137^Cs measured in this study were all at background levels, which were lower than those recommended by WHO.

Mean annual activity concentrations of ^90^Sr in different types of food decrease according to rice < cabbage < mullet < crucian carp. The mean *AEDs* of local food containing ^90^Sr and ^137^Cs for all three age groups in Sanmen County from 2011 to 2020 were significantly lower than the threshold recommended by the local government. Therefore, radioactivity concentrations of the investigated food products in Sanmen County (China) were at safe levels during 2011–2020.

The ^90^Sr and ^137^Cs radioactive materials detected in water and food samples in the study area were mainly from the global fallout of nuclear weapons tests. Since the operation of SNPP in 2018 until 2020, there has been no notable release of radioactive material to the study area.

## Data availability statement

The original contributions presented in the study are included in the article/supplementary material, further inquiries can be directed to the corresponding author.

## Ethics statement

Ethical review and approval was not required for the study on human participants in accordance with the local legislation and institutional requirements. Written informed consent from the participants was not required to participate in this study in accordance with the national legislation and the institutional requirements.

## Author contributions

PW and YC: study design, collection, analysis, interpretation of data, and final approval of the manuscript. SY, HZ, XL, HR, LZ, ZL, ZX, XG, QX, ZZ, and YZ: study design, collection, detection, analysis, interpretation of data, and manuscript writing. All authors contributed to the article and approved the submitted version.
